# How Is Connectedness With Nature Linked to Life Satisfaction or Depression Among Chinese People Living in Rural Low-Income Households? A Serial Mediation Model

**DOI:** 10.3389/fpubh.2022.827046

**Published:** 2022-04-29

**Authors:** Chunyu Yang, Xiaoyan Chen, Jun Yao, Jing An

**Affiliations:** ^1^Research Institute of Climatic and Environmental Governance, School of Law and Political Science, Nanjing University of Information Science and Technology, Nanjing, China; ^2^School of Humanities, Jiangsu University of Technology, Changzhou, China; ^3^School of Health Policy and Management, Nanjing Medical University, Nanjing, China; ^4^School of Management, Nanjing University of Posts and Telecommunications, Nanjing, China

**Keywords:** connectedness to nature, life satisfaction, depression, affect balance, social cohesion

## Abstract

**Objectives:**

In this study a serial multiple mediation model is tested to investigate the potential sequentially-mediating effect of affect balance and social cohesion on the association between connectedness to nature and life satisfaction or depression.

**Methods:**

A total of 675 Chinese people from Jiangsu province living in rural low-income households participated in the study. The Connectedness to Nature Scale (CNS), the Positive Affect and Negative Affect Scale (PANAS), the Social cohesion scale (SCS), the Satisfaction with Life Scale (SWLS), and the Patient Health Questionnaires (PHQ9) were measured in this paper.

**Results:**

Results indicated that the multiple serial mediation of affect balance and social cohesion on the association between connectedness to nature and life satisfaction was significant among the full-size sample, the adult-report, and the old people report, but that this effect is relatively small. Specifically, serial mediation accounted for 2.01, 1.69, 2.67% of the total effect explained by connectedness to nature on life satisfaction, while it accounted for 2.66, 2.35, 2.91% of the total effect explained by connectedness to nature on depression among the full sample population, adults, and old people, respectively.

**Conclusions:**

The findings corroborate the important roles of affect balance and social cohesion in activating connectedness to nature. We discussed the possible ways that affect balance and social cohesion might enhance life satisfaction and decrease depression for Chinese people living in rural low-income households. We also discussed the limitations of this study. More mechanisms could be considered in future studies.

## Introduction

There is a long-theorized history of investigation by ecologists and ecopsychologists of the relationship between human psychological relationships and the natural world. An early theme of those studies is that connectedness to nature serves as an important component impacting ecological behavior (1, 2). After reading Leopold's work, Mayer and Frantz (3) designed the Connectedness to Nature scale (CNS) that has been widely used in the areas of environmental and social psychology.

Mayer and Frantz (3) defined connectedness with nature as “an individual's experiential sense of oneness with the natural world”, and many studies have further discussed the connection between connectedness with nature and pro-environmental attitude and behavior (4), and underlying mechanisms of this relationship (5). Many authors have also found CNS to be firmly related to psychological health (6, 7) and psychological restoration (8, 9). To further investigate the underlying mechanisms of the relationship between CNS and psychological health, this study integrated affect balance and social cohesion as mediators in the associations between life satisfaction and depression among individuals living in low-income households in rural China.

## Connectedness To Nature, Affect Balance, Life Satisfaction and Depression

Many theoretical and empirical research studies argue that CNS is an instinctive human need providing psychological benefits positively related to various positive emotion indices, including positive affect (10), life satisfaction (3), and well-being (11). At the same time, connectedness to nature has been found to decrease individuals' negative outcomes, including distress, anxiety, stress, and depression (9, 12).

A biophilia perspective proposed by Wilson (13) suggests that this might be because individuals tend to seek out exposure to nature and may spend considerable time outdoors immersed in nature, providing them with opportunities to interact with other living things, thereby helping them avoid negative affect and become psychologically satisfied (10, 14). On the other hand, taking the perspective of the Eco-Existential Positive Psychology (EEPP), Passmore and Howell (15) asserted that an individual's sense of CNS may take a key role in addressing existential anxieties such as happiness, isolation, identity, freedom, and death, encouraging people to become part of the natural world, leading to an eco-centered view and lifestyle. This helps individuals with a stronger sense of CNS that makes them more likely to enjoy nature and to pay more attention to nature, resulting in more positive psychology (16) and enhancing recovery from stress and other negative outcomes (17–19).

Affect balance is hypothesized to be one mediator between CNS and life satisfaction or depression. So far we know, there has been little research that directly discusses the relationship between CNS and affect balance, defined as the balance of positive and negative affect (20, 21), or between affect balance and life satisfaction or depression. There are well-documented research studies that have found that CNS could promote positive affect and thereby increase life satisfaction (10, 22) while decreasing negative affect, thereby decreasing people's depression (9, 12). We therefore propose Hypotheses I and II as:

*Hypotheses I:* Connectedness to nature is positively related to life satisfaction, while negatively related to depression.

*Hypothesis II*: Affect balance takes mediating role in the association between connectedness to nature and life satisfaction or depression.

## Connectedness To Nature, Neighborhood Social Cohesion, Life Satisfaction and Depression

Neighborhood social cohesion is hypothesized to be another mediator between CNS and life satisfaction or depression. According to the self-determination theory proposed by Ryan and Deci (23), autonomy, competence, and relatedness are three basic psychological needs for enhancement of psychological growth, integrity, and well-being. Among these, the relatedness of basic psychological needs may lead individuals to increased feelings of CNS (24), with nature connection promoting a distinct form of relatedness with social connectedness (25) and playing a significant role in predicting happiness. Many research studies have also demonstrated that social cohesion could improve an individual's life satisfaction (26–28). As the term person-environment fit suggests, when personal beliefs, goals, abilities, and needs of an individual fit together with their social environment, individuals find it easier to pursue and accomplish their goals, thereby improving their well-being (29).

Many studies have also indicated that social cohesion is related to depression, with higher levels of social cohesion related to occurrence of fewer depressive symptoms (30). For example, a lower level of neighborhood-related social cohesion would tend to increase neighborhood disorder, thus increasing an individual's stress, anxiety, and depression (30, 31). A recent Mata-and Pooled analyses suggested that lack of social cohesion was significantly associated with depression among adults aged 50 years or older from 16 high-income countries (32).

There is also a lack of research related to whether serial mediation effects achieved via affect balance and neighborhood social cohesion exists in the relationship between CNS and life satisfaction or depression. However, in a meta-analysis of the relationship between CNS and happiness, Capaldi et al. (33) reported that considering positive emotional and social bonds as mediators in this relationship would offer a valuable extension to current research, and might offer support for Fredirkckson's (34) broaden-and-built theory, which asserts that psychological adaption evolved with positive emotion could have increased human ancestors' odds of survival and reproduction (35). Therefore, we propose Hypotheses 3 and Hypotheses 4 as:

*Hypothesis III*: Neighborhood social cohesion plays a mediating role in the association between connectedness to nature and life satisfaction or depression.

*Hypothesis IV*: A serial mediation effect exists in the relationship between connectedness to nature and life satisfaction and depression achieved via affect balance and social cohesion.

## The Present Study

**Figure 1:**
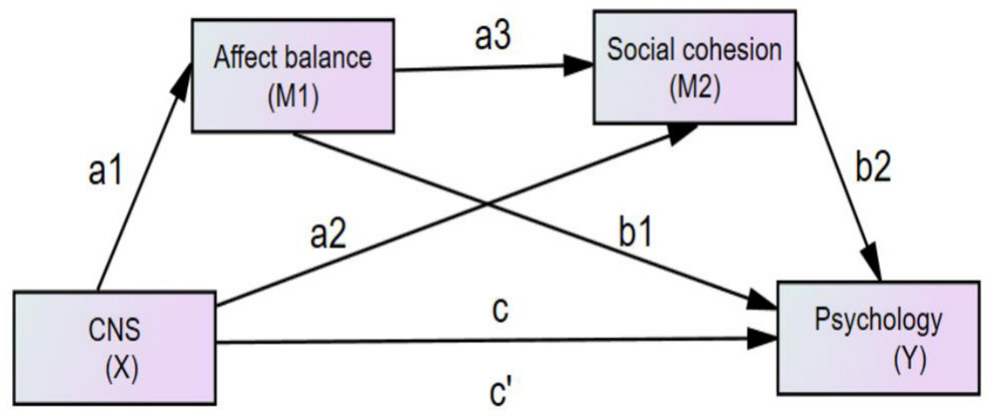
The hypothesized model concerning the relationship between connectedness to nature and psychological adjustment: affect balance and social cohesion as mediators. CNS(X), connectedness to nature; Psychology(Y), life satisfaction or depression; *c*′, the direct effect from X to Y; c, total effect from X to Y.

This study is designed to test for additional mechanisms in the association between CNS and life satisfaction or depression. According to Hayes (36), through mediating variables. a mediation model could partition the total effect of an independent variable on a dependent variable into direct and indirect effects in the association between connectedness to nature and life satisfaction or depression. In this study we specifically used serial multiple mediation. To test our hypotheses and determine whether the results would be the same among different populations, we utilized four models yielding two outcomes (life satisfaction and depression) and two questionnaire-based reports, an adult report (ages from 18 to 59 years) and an older people report (aged over 60 years). We also hypothesized that affect balance and social cohesion are two mediators in the association between connectedness to nature and life satisfaction or depression; the hypothesized model is shown in Figure 1.

## Materials and Methods

### Participants and Data Collection Procedure

A total of 675 Chinese individuals, comprised of 380 males, 293 females, and 2 persons not reporting their gender, all living in rural low-income households from Jiangsu province, participated in the study without compensation. All participants provided informed consent before the investigation and independently completed the face-to-face questionnaire process that took approximately 45 minutes.

### Measures

#### Connectedness to Nature

A 14-item version of the Connectedness to Nature Scale (CNS) developed by Mayer and Frantz (3) was used to measure individual ‘levels of feeling emotionally-connected to the natural world', i.e., their sense of oneness with the natural world. Items were rated on 5-point scales ranging from 1 (strongly disagree) to 5 (strongly agree), producing total scores between 5 and 70, with higher scores denoting a greater sense of nature connectedness. The CNS differs somewhat from other similar scales, such as the new environmental paradigm scale (NEP), the inclusion of nature in the self scale (INS), or the implicit associations test (IAT). Specifically, the CNS scale is affective (unlike NEP and Schults' related conception connectedness to nature scale), reliable, multi-item (unlike INS), easy to administer, and predicts behavior quite satisfactorily (unlike IAT). In this study, the Cronbach alpha coefficient of the scale was 0.856.

#### Affect Balance

Watson et al. (37) Positive Affect and Negative Affect Scale (PANAS) was used to measure both individual positive affect and negative affect. The scale consists of 20 items, including 10 positive-affect items and 10 negative-affect items. Affect balance, positive affect, and negative affect scores would be generated for each respondent, with the affect balance score the difference between positive affect and negative affect. Items were rated on a 5-point Likert scale ranging from 1 (very slight or not at all) to 5 (many), with higher scores denoting higher levels of affect balance. In this study, the Cronbach alpha coefficient of the scale was 0.791.

#### Neighborhood Social Cohesion

Neighborhood social cohesion was measured using a short version of the social cohesion and trust scale developed by Sampson et al. (38), based on 5-point Likert items ranging from 1 (strongly agree) to 5 (strongly disagree). The scale consisted of 4 questions including whether their neighborhoods are: willing to help each one another, get along well, be trusted, and “close knit.” Positively-worded items were reverse-coded, i.e., higher scores represented greater degrees of social cohesion. In this study, the Cronbach alpha coefficient of the scale was 0.936.

#### Life Satisfaction

Diener et al.'s (39) Satisfaction with Life Scale (SWLS) was used to measure an individual's life satisfaction. The scale consists of 5 items rated on a 7-point Likert scale ranging from 1 (strongly disagree) to 7 (strongly agree), with higher scores denoting higher levels of life satisfaction. The Cronbach's alpha of SWLS in this study was 0.932.

#### Patient Health Questionnaires (PHQ9)

Spitaer et al. (40) Patient Health Questionnaire (PHQ) is a brief diagnostic tool for evaluating depression. The scale consists of 9 items rated on a 4-point Likert scale ranging from 1 (not at all) to 4 (nearly every day), with higher scores denoting higher levels of depression. The Cronbach alpha coefficient of the Chinese version of the scale was 0.878.

### Statistical Analyses

We utilized SPSS statistical software (Version 24.0) to perform bivariate correlations among the study variables. Alpha, a number lying between 0 and 1, is used as a measure of a test or scale's internal consistency (41). Normally, acceptable alpha values are greater than 0.6, with a higher number indicating higher consistency. Moreover, in accordance with Hayes (36), we used the SPSS PROCESS macro from Model 6 to test the serial multiple mediation effect of affect balance and social cohesion in the relationship between connectedness to nature and psychological adjustment. If the indirect effect in the pathway connectedness to nature → affect balance → social cohesion → life satisfaction/depression was significant, we could conclude there was existence of serial multiple mediation, and could also calculate the percentage of each mediator in the total effect though division.

Furthermore, in accordance with to Hayes and Scharkow (42), we used bootstrapping used to extract 10,000 samples from the original data set (*n* = 387 for adult models, and 288 for old people models) via random sampling, with the mediation effect considered significant at the 0.05 level if the 95% CI (confidence interval) of the outcome of the mediation effect did not contain zero. Finally, to distinguish between adults and old people, we generated a psychological adjustment factor using life satisfaction and depression scores as indices, with self-reported outcomes for adult and old people tested separately.

## Results

### Demographic Information

Participants' ages ranged from 21 to 92 years old. For the education variable, number of years was considered as a reference criterion: variable “1 = below elementary school” comprised the largest proportion (*P* = 37.6%), while variable “5 = college and above” comprised the smallest proportion (*P* = 3.7%). For marital status, “married” accounted for 81.8% (*n* = 551) of the participants, while “single”, “divorced”, and “widowed” comprised 6.8% (*n* = 46), 1.6% (*n* = 11), and 9.6% (*n* =65) of the respondents, respectively. Furthermore, 46.7% of the participants reported annual incomes below 3,000 yuan, 25.6% between 3,000 and 6,000 yuan, and 13.9% more than 10,000 yuan (see Table 1).

**Table 1:** Sample characteristics (olds: ≥60).

**Sample characteristics**		**Total (*N* = 675)**		**Adult (*N* = 288)**		**Old (*N* = 386)**	
		**M**	**SD**	**M**	**SD**	**M**	**SD**
		***n***	**%**	***n***	**%**	***n***	**%**
**Age (21-92 years): (*****n** **=*** **674)**		60.19	12.03	48.91	8.13	68.6	6.13
**Gender: (*****n** **=*** **673)**	1. Male	380	56.3	125	43.4	254	65.8
	2. Female	293	43.4	163	56.6	130	33.7
**Education: (*****n** **=*** **674)**	1. Below Elementary school	254	37.6	74	25.7	180	46.6
	2. Elementary school	192	28.4	92	31.9	99	25.6
	3. Middle school	156	23.1	77	26.7	79	20.5
	4. High school	51	7.6	23	8.0	28	7.3
	5.College and above	21	3.1	21	7.3	0	0
**Marital status: (*****n** **=*** **674)**	1.Single	46	6.8	21	7.3	25	6.5
	2.Married	551	81.8	250	86.8	300	77.7
	3.Divorced	11	1.6	8	2.8	3	0.8
	4.Widowed	65	9.6	8	2.8	57	14.8
	5.Others	1	0.1	0	0	1	0.3
**Annually income (yuan/year): (*****n** **=*** **650)**	<3,000	315	46.7	109	37.8	206	53.4
	3,000–6,000	173	25.6	73	25.3	100	25.9
	6,000–10,000	68	10.1	31	10.8	37	9.6
	>10,000	94	13.9	68	23.6	25	6.5

### Bivariate Associations

Table 2 shows the mean, SD, alpha, and the bivariate correlations of all the variables considered in this study. These results showed significant correlations among all the variables, consistent with expectations. Moreover, connectedness to nature was positively-related to affect balance, social cohesion, and life satisfaction, while depression was negatively-related to connectedness to nature, affect balance, social cohesion, and life satisfaction.

**Table 2:** Means, standard deviations (SD), alpha, reliabilities and intercorrelations among study variables.

**Measure**	**Mean**	**SD**	**α**	**1**	**2**	**3**	**4**	**5**
1. Connectedness to nature	51.39	7.54	0.856	1				
2. Affect balance	2.08	5.24	0.791	0.253^**^	1			
3. Social cohesion	17.54	2.51	0.936	0.267^**^	0.195^**^	1		
4. Life satisfaction	24.05	7.94	0.932	0.283^**^	0.402^**^	0.281^**^	1	
5. Depression	14.91	5.66	0.878	−0.230^**^	−0.494^**^	−0.282^**^	−0.472^**^	1

*Sample sizes: n = 675*.

*α, Cronbach's alpha*.

** *Correlation is significant at the 0.01 level (2-tailed)*.

### Serial Multiple Mediation Model

**Figure 2:**
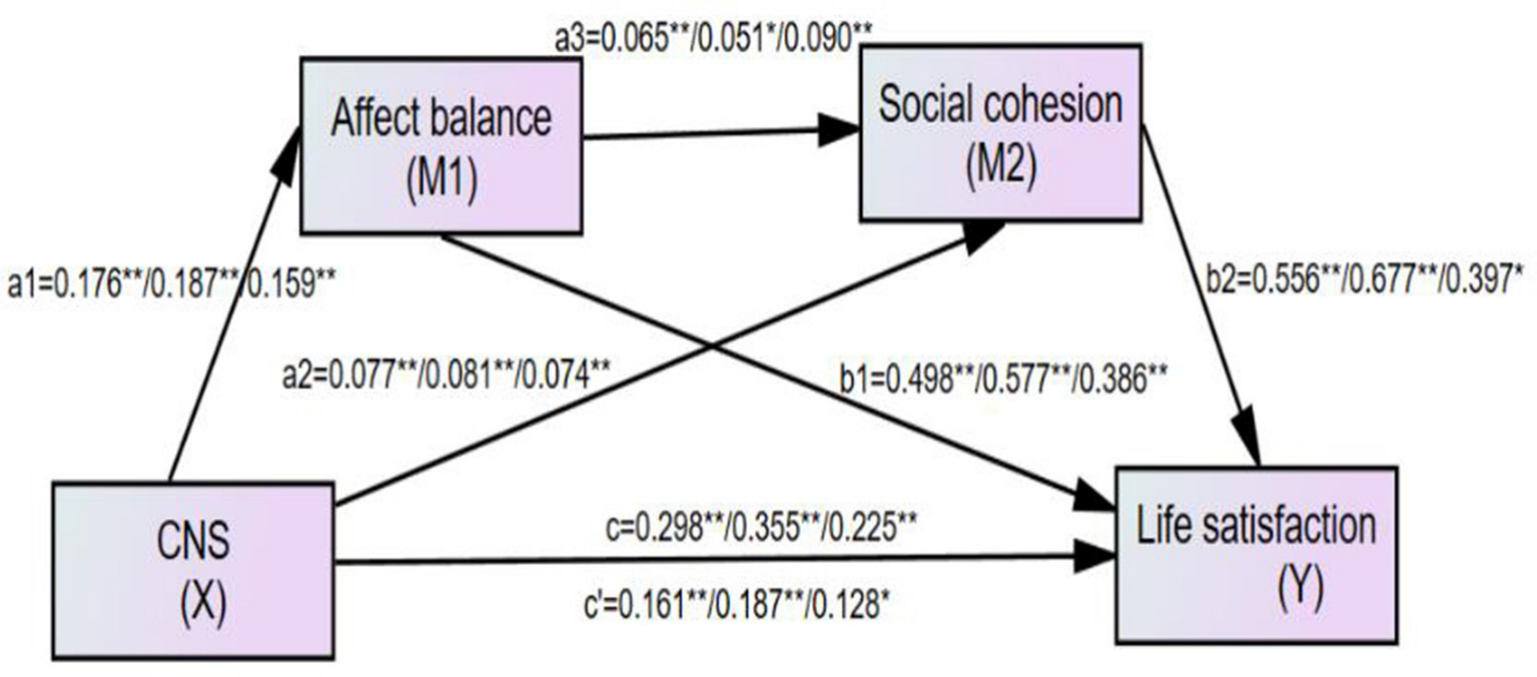
The finalized structural model concerning the relationship between connectedness to nature and life satisfaction: affect balance and social cohesion as mediators. Left numbers, the full sample (*N* = 675); Middle number, the adult sample (*N* = 386); Right number, the old people sample (*N* = 288); *c*′, the direct effect from connectedness to nature to life satisfaction; *c*, total effect from connectedness to nature to life satisfaction. **Correlation is significant at the 0.01 level. *Correlation is significant at the 0.05 level.

**Figure 3:**
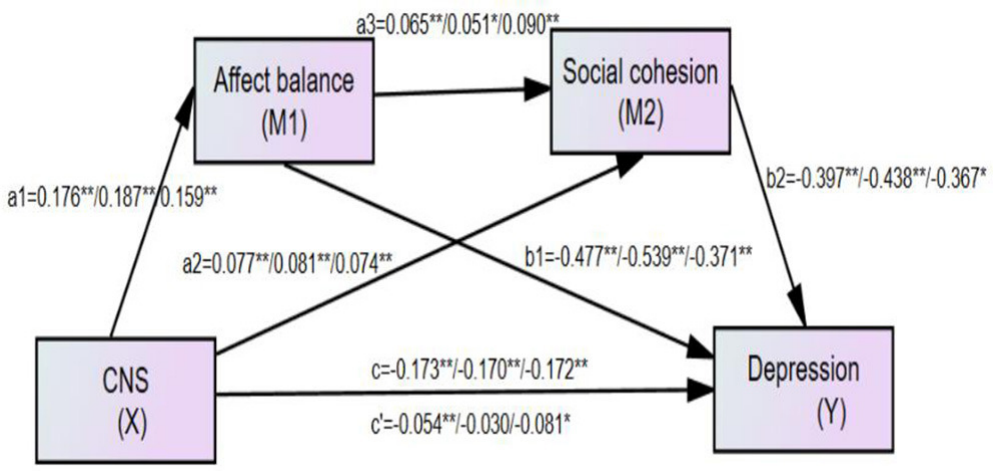
The finalized structural model concerning the relationship between connectedness to nature and depression: affect balance and social cohesion as mediators. Left numbers, the full sample (*N* = 675); Middle number, the adult sample (*N* = 386); Right number, the old people sample (*N* = 288); *c*′, the direct effect from connectedness to nature to depression; c, total effect from connectedness to nature to depression. **Correlation is significant at the 0.01 level. *Correlation is significant at the 0.05 level.

We used a serial multiple mediation model specified by Hayes (36) to test whether the effect of connectedness to nature on life satisfaction or depression is mediated serially, moving from connectedness to nature (X) to affect balance (M1) to social cohesion (M2) to life satisfaction/depression (Y). Specifically, two outcomes (life satisfaction and depression) against adult-report and old people-report were measured in separate serial multiple mediation models, with the results shown in Table 3. Life satisfaction score was tested by the first mediation model, and results of all path coefficients for the full sample (left number), the adult sample (middle number), and the old-people sample (right number), are shown in Figure 2, respectively, in to the first, third, and fifth columns of Table 3. Moreover, the Depression score was tested by the second mediation model, and results of all path coefficients for the full sample (left number), the adult sample (middle number), and the old-people sample (right number), are shown in Figure 3, respectively, in the second, fourth, and sixth columns of Table 3.

**Table 3:** Path coefficients and standard errors from serial mediation models estimated using process.

**Path**	**Full sample (*n* = 675)**		**Adult sample (*n* = 386)**		**Old people sample (*n* = 288)**	
	**Life satisfaction**	**Depression**	**Life satisfaction**	**Depression**	**Life satisfaction**	**Depression**
**Total effect (c)**	0.298^**^ (0.039)	−0.173^**^ (0.028)	0.355^**^ (0.052)	−0.170^**^ (0.041)	0.225^**^ (0.060)	−0.172^**^ (0.036)
95% CI	[0.222, 0.375]	[−0.228, −0.118]	[0.254, 0.457]	[−0.251, −0.090]	[0.108, 0.342]	[−0.244, −0.101]
**Direct effect (** ***c** **′*** **)**	0.161^**^ (0.038)	−0.054^**^ (0.028)	0.187^**^ (0.049)	−0.030 (0.038)	0.128^*^ (0.060)	−0.081^*^ (0.034)
95% CI	[0.087, 0.236]	[−0.105, −0.003]	[0.091, 0.282]	[−0.104, 0.044]	[0.010, 0.247]	[−0.148, −0.144]
X → M1 (*a*_1_)	0.176^**^ (0.026)	0.176^**^ (0.026)	0.187^**^ (0.035)	0.187^**^ (0.035)	0.159^**^ (0.038)	0.159^**^ (0.038)
95% CI	[0.125, 0.227]	[0.125, 0.227]	[0.117, 0.256]	[0.117, 0.256]	[0.084, 0.234]	[0.084, 0.234]
X → M2 (*a*_2_)	0.077^**^ (0.013)	0.077^**^ (0.013)	0.081^**^ (0.017)	0.081^**^ (0.017)	0.074^**^ (0.019)	0.074^**^ (0.019)
95% CI	[0.053, 0.102]	[0.053, 0.102]	[0.047, 0.114]	[0.047, 0.114]	[0.036, 0.113]	[0.036, 0.113]
M1 → M2 (*a*_3_)	0.065^**^ (0.018)	0.065^**^ (0.018)	0.051^*^ (0.024)	0.051^*^ (0.024)	0.090^**^ (0.029)	0.090^**^ (0.029)
95% CI	[0.030, 0.101]	[0.030, 0.101]	[0.004, 0.098]	[0.004, 0.098]	[0.033, 0.146]	[0.033, 0.146]
M1 → Y (*b*_1_)	0.498^**^ (0.054)	−0.477^**^ (0.037)	0.577^**^ (0.066)	−0.539^**^ (0.051)	0.386^**^ (0.090)	−0.371^**^ (0.051)
95% CI	[0.3920, 0.6029]	[−0.549, −0.405]	[0.447, 0.707]	[−0.640, −0.439]	[0.209, 0.563]	[−0.471, −0.270]
M2 → Y(*b*_2_)	0.556^**^ (0.077)	−0.397^**^ (0.077)	0.677^**^ (0.141)	−0.438^**^ (0.109)	0.397^*^ (0.183)	−0.367^*^ (0.104)
95% CI	[0.335, 0.777]	[−0.5480, −0.246]	[0.399, 0.955]	[−0.653, −0.224]	[0.037, 0.757]	[−0.571, −0.164]
**Indirect effects**						
X → M1 → Y (*a*_1_*b*_1_)	0.088^**^ (0.017)	−0.084^**^ (0.017)	0.108^**^ (0.027)	−0.101^**^ (0.025)	0.062^**^ (0.022)	−0.059^**^ (0.018)
95% CI	[0.057, 0.127]	[−0.121, −0.055]	[0.061, 0.168]	[−0.155, −0.057]	[0.048, 0.161]	[−0.102, −0.028]
X → M2 → Y (*a*_2_*b*_2_)	0.043^**^ (0.012)	−0.031^**^ (0.009)	0.055^**^ (0.016)	−0.035^**^ (0.013)	0.029^**^ (0.017)	−0.027^**^ (0.010)
95% CI	[0.023, 0.070]	[−0.051, −0.017]	[0.029, 0.091]	[−0.069, −0.015]	[0.002, 0.071]	[−0.053, −0.011]
X → M1 → M2 → Y (*a*_1_*a*_3_*b*_2_)	0.006^**^ (0.003)	−0.0046^**^ (0.002)	0.006^**^ (0.004)	−0.004^**^ (0.002)	0.006^**^ (0.004)	−0.005^**^ (0.003)
95% CI	[0.002, 0.013]	[−0.009, −0.002]	[0.004, 0.017]	[−0.010, −0.001]	[0.001, 0.017]	[−0.014, −0.001]
**Total indirect effects**	0.137^**^ (0.021)	−0.119^**^ (0.018)	0.169^**^ (0.030)	−0.140^**^ (0.026)	0.097^**^ (0.028)	−0.091^**^ (0.023)
95% CI	[0.099, 0.182]	[−0.1583, −0.0868]	[0.114, 0.223]	[−0.194, −0.093]	[0.048, 0.161]	[−0.143, −0.052]
R^2^	0.080	0.053	0.110	0.043	0.048	0.073
F	58.65	37.70	47.574	17.23	14.254	22.53
P	<0.001	<0.001	<0.001	<0.001	<0.001	<0.001

*Sample sizes: for connectedness to nature, life satisfaction, depression, full sample = 675, adult sample n = 386, old people sample n = 288*.

*X, Connectedness to nature; M1, Affect balance; M2, Social cohesion; Y, life satisfaction or depression*.

** *Correlation is significant at the 0.01 level*.

* *Correlation is significant at the 0.05 level*.

### Full-Sample Data: Life Satisfaction and Depression

Full-sample results indicated that the total effect of connectedness to nature on life satisfaction was significant (*c* = 0.298, *p* < 0.001, 95% CI [0.222, 0.375]), supporting our hypothesis that affect balance and social cohesion serially mediate the association between connectedness to nature and life satisfaction. The total indirect effect of affect balance and social cohesion was significant (total indirect effect = 0.137, *p* < 0.01, 95% CI [0.099, 0.182]), accounting for 45.97% of the total effect, while the total direct effect was also significant (*c*′ = 0.161, *p* < 0.01, 95% CI [0.087, 0.236]). The results also showed that the serial mediation model was supported, because in the path “connectedness to nature → affect balance → social cohesion → life satisfaction”, the serial indirect effect was found to be significant (*a*_1_*a*_3_*b*_2_ = 0.006, 95% CI [0.002, 0.070]), accounting for 2.01% of the total effect. Furthermore, in the path “connectedness to nature → affect balance → life satisfaction”, the indirect effect of affect balance was significant (*a*_1_*b*_1_ = 0.088, 95% CI [0.057, 0.127]), while in the path “connectedness to nature → social cohesion → life satisfaction”, the indirect effect of social cohesion was also significant (*a*_2_*b*_2_ = 0.043, 95% CI [0.023, 0.070]), accounting for 29.5 and 14.4%, respectively, of the total effect of connectedness to nature on life satisfaction.

The full-sample results indicated that the total effect of connectedness to nature on depression was significant (*c* = −0.173, *p* < 0.001, 95% CI [−0.228, −0.118]), supporting our hypothesis that affect balance and social cohesion serially mediate the association between connectedness to nature and depression. The total indirect effect of affect balance and social cohesion was significant (total indirect effort = −0.119, *p* <, 95% CI [−0.1583, −0.086]), accounting for 68.79% of the total effect, while the total direct effect was also significant (*c*′ = −0.054, *p* < 0.001, 95% CI [−0.105, −0.003]). The results also showed that the serial mediation model was supported; in the path “connectedness to nature → affect balance → social cohesion → depression” the serial indirect effect was significant (*a*_1_*a*_3_*b*_2_ = −0.0046, 95% CI [−0.009, −0.002]), accounting for 2.66% of the total effect. Furthermore, in the path “connectedness to nature → affect balance → depression”, the indirect effect of affect balance was significant (*a*_1_*b*_1_ = −0.084, 95% CI [−0.121, −0.055]), while in the path “connectedness to nature → social cohesion → depression”, the indirect effect of social cohesion was also significant (*a*_2_*b*_2_ = −0.031, 95% CI [−0.051, −0.017]), accounting for 48.6 and 17.9%, respectively, of the total effect of connectedness to nature on depression.

### Adult-Report Data: Life Satisfaction and Depression

Life satisfaction in the adult sample was also tested, with the results shown in Figure 2 (middle number) and the third column of Table 3. The total effect was significant (*c* = 0.355, *p* < 0.001, 95% CI [0.254, 0.457]), and the total indirect effect was also significant (total indirect effect = 0.169, *p* < 0.01, 95% CI [0.114, 0.223]), accounting for 47.61% of the total effect). The direct effect of connectedness to nature on life satisfaction was also significant (*c*′ = 0.187, *p* < 0.001, 95% CI [0.091, 0.282]). Moreover, the serial-mediation hypothesis was supported (*a*_1_*a*_3_*b*_2_ = 0.006, 95% CI [0.004, 0.017]), and each indirect effect was significant (*a*_1_*b*_1_ = 0.108, 95% CI [0.061, 0.168]; *a*_2_*b*_2_ = 0.055, 95% CI [0.029, 0.091]).

Depression in the adult-sample was also tested, with the results shown in Figure 2 (middle number) and the fourth column of Table 2. The total effect was significant (*c* = −0.170, *p* < 0.001, 95% CI [−0.251, −0.090]), and the total indirect effect was also significant (total indirect effect = −0.140, *p* < 0.01, 95% CI [−0.194, −0.093]), accounting for 47.61% of the total effect. However, the direct effect of connectedness to nature on depression was not significant (*c*′ = −0.030, *p* > 0.05, 95% CI [−0.104, 0.044]). Moreover, the serial-mediation hypothesis was supported (*a*_1_*a*_3_*b*_2_ = −0.004, 95% CI [−0.010, −0.001]), and each indirect effect was significant (*a*_1_*b*_1_ = −0.101, 95% CI [−0.155, −0.057]; *a*_2_*b*_2_ = −0.035, 95% CI [−0.069, −0.015]).

### Old People-Report Data

The same analyses were performed for old people, and the results for life satisfaction and depression are shown in Figure 2 (right number), Figure 3 (right number), and the fifth and sixth columns of Table 3, respectively. The results indicated that either of the models supported our hypothesis with respect to both life satisfaction and depression model. Specifically, the serial mediation effect and each indirect effect were significant both in the life satisfaction model (a_1_a_3_b_2_ = 0.006, 95% CI [0.001, 0.017]; *a*_1_*b*_1_ = 0.062, 95% CI [0.048, 0.161]; *a*_2_*b*_2_ = 0.029, 95% CI [0.002, 0.071]) and the depression model (*a*_1_*a*_3_*b*_2_ = −0.005, 95% CI [−0.014, −0.001]; *a*_1_*b*_1_ = −0.059, 95% CI [−0.102, −0.028]; *a*_2_*b*_2_ = −0.027, 95% CI [−0.053, −0.011]). Moreover, the total indirect effect was significant in the life satisfaction model (total indirect effect = 0.097, *p* < 0.01, 95% CI [0.048, 0.161]) and the depression model (total indirect effect = −0.091, *p* < 0.01, 95% CI [−0.143, −0.052]), accounting for 43.11%, and 37.30% of the total effect in life satisfaction (*c* = 0.225, *p* < 0.001, 95% CI [0.108, 0.342]) and depression (*c* = −0.172, *p* < 0.001, 95% CI [−0.244, −0.101]), respectively.

### Supplemental Analyses

**Figure 4:**
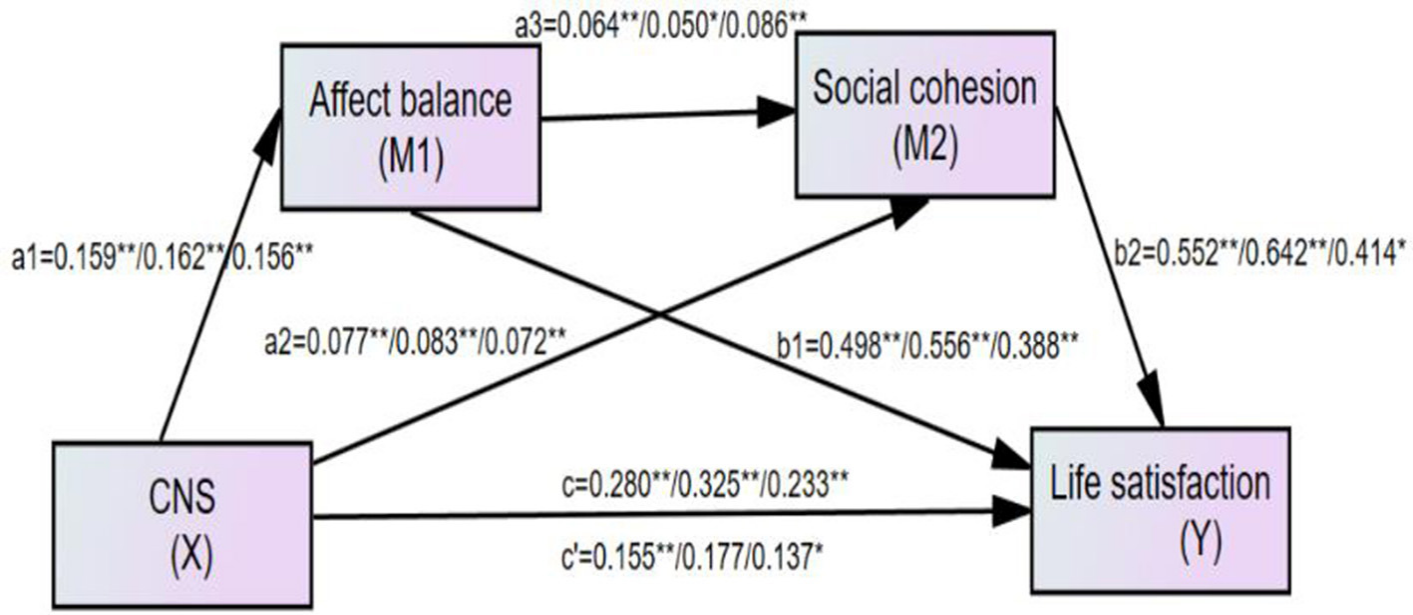
The finalized structural model after controlling gender, age, marriage status and education level concerning the relationship between connectedness to nature and life satisfaction: affect balance and social cohesion as mediators. Left numbers, the full sample (*N* = 670); Middle number, the adult sample (*N* = 384); Right number, the old people sample(*N* = 286); *c*′, the direct effect from connectedness to nature to life satisfaction; c, total effect from connectedness to nature to life satisfaction. **Correlation is significant at the 0.01 level. *Correlation is significant at the 0.05 level.

**Figure 5:**
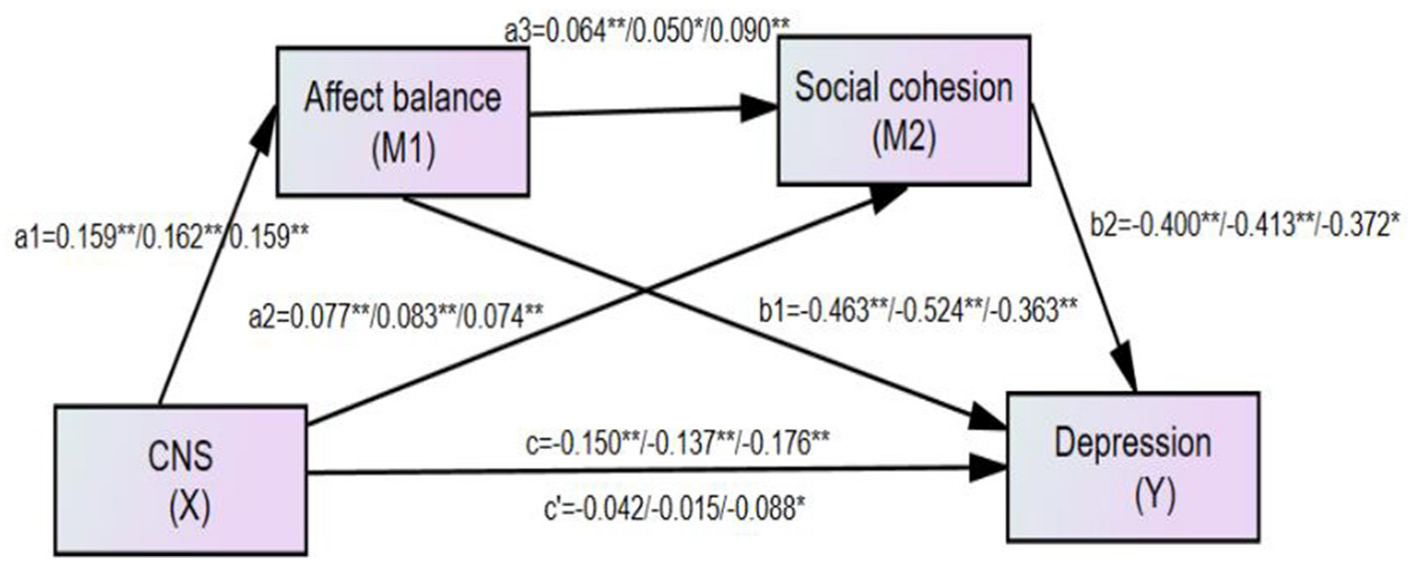
The finalized structural model after controlling gender, age, marriage status and education level concerning the relationship between connectedness to nature and depression: affect balance and social cohesion as mediators. Left numbers, the full sample (*N* = 670); Middle number, the adult sample (*N* = 384); Right number, the old people sample (*N* = 286); *c*′, the direct effect from connectedness to nature to depression; *c*, total effect from connectedness to nature to depression. **Correlation is significant at the 0.01 level. * Correlation is significant at the 0.05 level.

There might be some potential confounders in this mediation model, such as gender, income, marriage status, and education level. After controlling for age, gender, marriage status and education level, the results in Table 4 for the full sample-size adult sample and the old people sample with respect to both life satisfaction and depression can be seen. After controlling covariates he results remained substantively unchanged. For example, in the model with life satisfaction among adult sample (seen the middle number of Figure 4 and the third column of Table 4), both the total indirect effect (total indirect effect = 0.148, *p* < 0.01, 95% CI [0.094, 0.213]), and direct effect (*c*′ = 0.177, *p* < 0.01, 95% CI [0.222, 0.428]) were significant, with the total indirect effect accounting for 45.54% of the total effect (*c* = 0.325, *p* < 0.001, 95% CI [0.222, 0.428]). The serial-mediation hypothesis was supported (*a*_1_*a*_3_*b*_2_ = 0.005, 95% CI [0.003, 0.015]), and each indirect effect was significant. The same analyses were performed for the whole sample, adult sample, and old people, and the results for depression are shown in Figure 5.

**Table 4:** Path coefficients and standard errors after controlling gender, age, marriage status and education level from serial mediation models estimated using process.

**Path**	**Full sample (*n* = 670)^a^**		**Adult sample (*n* = 384)**		**Old people sample (*n* = 286)**	
	**Life satisfaction**	**Depression**	**Life satisfaction**	**Depression**	**Life satisfaction**	**Depression**
**Total effect (c)**	0.280^**^ (0.039)	−0.150^**^ (0.028)	0.325^**^ (0.052)	−0.137^**^ (0.041)	0.233^**^ (0.060)	−0.176^**^ (0.037)
95% CI	[0.203, 0.357]	[−0.205, −0.094]	[0.222, 0.428]	[−0.219, −0.056]	[0.114, 0.352]	[−0.249, −0.103]
**Direct effect (** ***c** **′*** **)**	0.155^**^ (0.038)	−0.042 (0.026)	0.177 (0.049)	−0.015 (0.038)	0.137^*^ (0.061)	−0.088^*^ (0.035)
95% CI	[0.0870, 0.230]	[−0.093, −0.0097]	[0.080, 0.273]	[−0.089, 0.059]	[0.017, 0.257]	[−0.156, −0.019]
X → M1 (a_1_)	0.159^**^ (0.026)	0.159^**^ (0.026)	0.162^**^ (0.036)	0.162^**^ (0.035)	0.156^**^ (0.039)	0.159^**^ (0.038)
95% CI	[0.107, 0.211]	[0.107, 0.211]	[0.090, 0.233]	[0.090, 0.233]	[0.079, 0.233]	[0.084, 0.234]
X → M2 (a_2_)	0.077^**^ (0.013)	0.077^**^ (0.013)	0.083^**^ (0.018)	0.083^**^ (0.018)	0.072^**^ (0.020)	0.074^**^ (0.019)
95% CI	[0.050, 0.101]	[0.050, 0.101]	[0.048, 0.117]	[0.048, 0.117]	[0.034, 0.111]	[0.036, 0.113]
M1 → M2 (a_3_)	0.064^**^ (0.018)	0.064^**^ (0.019)	0.050^*^ (0.024)	0.050^*^ (0.024)	0.086^**^ (0.029)	0.090^**^ (0.029)
95% CI	[0.028, 0.101]	[0.028, 0.101]	[0.002, 0.098]	[0.002, 0.098]	[0.029, 0.143]	[0.033, 0.146]
M1 → Y (b_1_)	0.489^**^ (0.054)	−0.463^**^ (0.037)	0.556^**^ (0.066)	−0.524^**^ (0.051)	0.388^**^ (0.090)	−0.363^**^ (0.051)
95% CI	[0.383, 0.593]	[−0.535, −0.391]	[0.426, 0.687]	[−0.624, −0.424]	[0.211, 0.564]	[−0.464, −0.262]
M2 → Y(b_2_)	0.552^**^ (0.111)	−0.400^**^ (0.077)	0.642^**^ (0.140)	−0.413^**^ (0.107)	0.414^*^ (0.182)	−0.372^*^ (0.105)
95% CI	[0.334, 0.770]	[−0.550, −0.250]	[0.367, 0.917]	[−0.624, −0.424]	[0.055, 0.772]	[−0.578, −0.166]
**Indirect effects**						
X → M1 → Y (*a*_1_*b*_1_)	0.078^**^ (0.017)	−0.073^**^ (0.017)	0.090^**^ (0.025)	−0.085^**^ (0.024)	0.065^**^ (0.021)	−0.057^**^ (0.019)
95% CI	[0.048, 0.115]	[−0.145, −0.077]	[0.046, 0.146]	[−0.173, −0.077]	[0.027, 0.113]	[−0.102, −0.026]
X → M2 → Y (*a*_2_*b*_2_)	0.042^**^ (0.012)	−0.030^**^ (0.009)	0.053^**^ (0.017)	−0.034^**^ (0.014)	0.030^**^ (0.018)	−0.027^**^ (0.010)
95% CI	[0.022, 0.012]	[−0.050, −0.016]	[0.026, 0.094]	[−0.068, −0.014]	[0.003, 0.074]	[−0.054, −0.011]
X → M1 → M2 → Y (*a*_1_*a*_3_*b*_2_)	0.006^**^ (0.003)	−0.0041^**^ (0.002)	0.005^**^ (0.004)	−0.003^**^ (0.002)	0.006^**^ (0.004)	−0.005^**^ (0.003)
95% CI	[0.002, 0.012]	[−0.008, −0.0017]	[0.003, 0.015]	[−0.0088, −0.0005]	[0.007, 0.017]	[−0.014, −0.001]
**Total indirect effects**	0.125^**^ (0.021)	−0.108^**^ (0.017)	0.148^**^ (0.030)	−0.1222^**^ (0.025)	0.096^**^ (0.028)	−0.089^**^ (0.024)
95% CI	[0.088, 0.169]	[−0.145, −0.077]	[0.094, 0.213]	[−0.173, −0.077]	[0.048, 0.161]	[−0.143, −0.048]
R^2^	0.099	0.073	0.137	0.082	0.064	0.276
F	14.73	10.49	11.96	6.712	3.809	15.17
P	<0.001	<0.001	<0.001	<0.001	<0.001	<0.001

*Sample sizes: for connectedness to nature, life satisfaction, depression, full sample n = 675, adult sample n = 386, old people sample n = 288*.

a *Sample sizes reduced for this process. Full sample = 670, adult sample n = 384, old people sample n = 286*.

*X, Connectedness to nature; M1, Affect balance; M2, Social cohesion; Y, life satisfaction or depression*.

** *Correlation is significant at the 0.01 level*.

* *Correlation is significant at the 0.05 level*.

## Discussion

Based on previous literature, we investigated the mediating role among low-income rural people in China of affect balance and social cohesion on the association between connectedness to nature and life satisfaction or depression. We hypothesized that connectedness to nature was positively related to life satisfaction, affect balance and social cohesion, and negatively associated with depression, and the results supported our Hypothesis I. Based on both adult-report and old people-report data, the results showed that connectedness to nature was related to higher levels of life satisfaction and lower levels of depression, a relationship that mediated by affect balance and social cohesion. Moreover, serial mediation accounted for 2.01, 1.69, 2.67% of the total effect, explained by connectedness to nature on life satisfaction, while among the full sample size, 2.66, 2.35, 2.91% of the total effect was explained by connectedness to nature on depression, adults, and old people, respectively. In all, the three indirect effects accounted for 45.97, 47.61, 43.11% of the total effect of connectedness to nature on life satisfaction, while accounting for 68.79, 82.35, 52.91% of the total effect of connectedness to nature on life satisfaction among full sample size, adults, and old people, respectively. Individuals with higher CNS scores could conceivably exhibit better emotion regulation, easier acceptance by others, and stronger feelings of social connectedness, thus achieving greater life satisfaction, relatively free from feelings of depression.

The findings of the old people-report data are consistent with those for the full sample size and the adult-report data. Affect balance and social cohesion played a mediating role in the association between connectedness to nature and life satisfaction. Individuals with higher levels of connectedness to nature tend to achieve greater affect balance and social cohesion and thereby enhance their life satisfaction. We can learn from these results that there are many ways to increase life satisfaction among low-income rural people in China. We can enhance the affect balance or social cohesion of low-income rural people in China to heighten their connectedness to nature level, thereby increasing their life satisfaction. Previous literature has indicated that affect balance can increase connectedness to nature, thereby increasing life satisfaction. An individual achieving higher connectedness to nature might enjoy greater life satisfaction through perception of greater more social cohesion (26, 43–45). For example, Sommer (46) indicated that feelings connected to the natural world in tree planting programs might help individuals fulfill social-relatedness needs, enhancing their social connectedness, leading to enhanced statements of well-being.

The mediation analysis also indicated that the effect of connectedness to nature on depression is mediated via the simple aspects of affect balance and social cohesion. Specifically, our final model showed that the path of connectedness to nature → affect balance → social cohesion → depression was significant, showing that people with higher levels of connectedness to nature are prone to obtain more affect balance and social cohesion, in turn producing less sense of depression. Moreover, this path indicates that affect balance is a mediator between connectedness to nature and depression. The study demonstrated that affect balance mediated the association between connectedness to nature and depression. In terms of recent research, individuals who have more connectedness to nature may maintain more adaptive emotion regulation strategies that in turn might lead to a decline in their negative outcomes (47). This path also indicated that social cohesion mediated the relationship between connectedness to nature and depression. Social cohesion has been found to be an important factor in decreasing depression-related judgments (30, 48). These results support our hypothesis II, III and IV, and may complement clinical treatment to improve people's, especially older adults', mental health and enhance their quality of life.

However, this study had several limitations. First, it lacked a control group (e.g., city people). Second, data collected by questionnaires and scales in the form of “face-to-face” interviews might be influenced by subjectivity, and future studies might benefit from use of multi-methods to collect the information. Third, since cross-sectional research, not exploring causal relationship, was used in this study, experimental and longitudinal research methods could be considered in the future to study relationships among connectedness to nature, affect balance, social cohesion, life satisfaction and depression. Finally, other possible mediating factors such as mindfulness, resilience, or climate-change belief, could be used in future studies to explore relationships between connectedness to nature, life satisfaction, and depression.

## Conclusion

This study expanded our knowledge about the complicated interplay among connectedness to nature, affect balance, social cohesion, and life satisfaction/ depression in Chinese low-income rural people. The significant path from connectedness to nature through affect balance and social cohesion to life satisfaction/ depression was used in discussing the underlying mechanisms between connectedness to nature and life satisfaction/ depression. Our findings may provide valuable insights for improving mental health of low-income rural people. Enhancement of connectedness to nature can possibly be used as positive therapy in helping Chinese low-income rural people lower their depression levels and increase their level of life satisfaction. It might also represent positive therapy to help them enhance the effects of affect balance and social cohesion, leading to enhanced mental health.

## Data Availability Statement

The raw data supporting the conclusions of this article will be made available by the authors, without undue reservation.

## Ethics Statement

Ethics approval and written informed consent were not required for this study in accordance with national guidelines and local legislation.

## Author Contributions

CY, XC, and JY drafted and conducted the manuscript. CY contributed to introduction, literature review, data analysis, and results and finalized the manuscript. XC and JY participated in conducted and polished the manuscript. JA contributed in the review process. All authors read and approved the final manuscript.

## Funding

This article was supported by National Social Science Foundation of China “A follow-up study on the influence mechanism of intergenerational relationship on the mental health of migrant elderly” (18BRK026), the Startup Foundation for Introducing Talent of NUIST (2021r064), and the National Natural Science Foundation of China (42001169).

## Conflict of Interest

The authors declare that the research was conducted in the absence of any commercial or financial relationships that could be construed as a potential conflict of interest.

## Publisher's Note

All claims expressed in this article are solely those of the authors and do not necessarily represent those of their affiliated organizations, or those of the publisher, the editors and the reviewers. Any product that may be evaluated in this article, or claim that may be made by its manufacturer, is not guaranteed or endorsed by the publisher.
